# *MKLN1* splicing defect in dogs with lethal acrodermatitis

**DOI:** 10.1371/journal.pgen.1007264

**Published:** 2018-03-22

**Authors:** Anina Bauer, Vidhya Jagannathan, Sandra Högler, Barbara Richter, Neil A. McEwan, Anne Thomas, Edouard Cadieu, Catherine André, Marjo K. Hytönen, Hannes Lohi, Monika M. Welle, Petra Roosje, Cathryn Mellersh, Margret L. Casal, Tosso Leeb

**Affiliations:** 1 Institute of Genetics, Vetsuisse Faculty, University of Bern, Bern, Switzerland; 2 DermFocus, University of Bern, Bern, Switzerland; 3 Department of Pathobiology, Institute of Pathology and Forensic Veterinary Medicine, University of Veterinary Medicine Vienna, Vienna, Austria; 4 Department of Small Animal Clinical Sciences, The University of Liverpool, Leahurst Campus, Neston, Cheshire, United Kingdom; 5 Antagene, Animal Genetics Laboratory, La Tour de Salvagny, France; 6 Institut de Génétique et Développement de Rennes (IGDR), CNRS-UMR6290, Université Rennes1, Rennes, France; 7 Department of Veterinary Biosciences, University of Helsinki, Helsinki, Finland; 8 Research Programs Unit, Molecular Neurology, University of Helsinki, Helsinki, Finland; 9 Folkhälsan Institute of Genetics, University of Helsinki, Helsinki, Finland; 10 Institute of Animal Pathology, Vetsuisse Faculty, University of Bern, Bern, Switzerland; 11 Division of Clinical Dermatology, Department of Clinical Veterinary Medicine, Vetsuisse Faculty, University of Bern,Bern, Switzerland; 12 Kennel Club Genetics Centre, Animal Health Trust, Kentford, Newmarket, Suffolk, United Kingdom; 13 Section of Medical Genetics, Department of Clinical Sciences & Advanced Medicine, School of Veterinary Medicine, University of Pennsylvania, Philadelphia, Pennsylvania, United States of America; University Medical Center Freiburg, GERMANY

## Abstract

Lethal acrodermatitis (LAD) is a genodermatosis with monogenic autosomal recessive inheritance in Bull Terriers and Miniature Bull Terriers. The LAD phenotype is characterized by poor growth, immune deficiency, and skin lesions, especially at the paws. Utilizing a combination of genome wide association study and haplotype analysis, we mapped the LAD locus to a critical interval of ~1.11 Mb on chromosome 14. Whole genome sequencing of an LAD affected dog revealed a splice region variant in the *MKLN1* gene that was not present in 191 control genomes (chr14:5,731,405T>G or *MKLN1*:c.400+3A>C). This variant showed perfect association in a larger combined Bull Terrier/Miniature Bull Terrier cohort of 46 cases and 294 controls. The variant was absent from 462 genetically diverse control dogs of 62 other dog breeds. RT-PCR analysis of skin RNA from an affected and a control dog demonstrated skipping of exon 4 in the *MKLN1* transcripts of the LAD affected dog, which leads to a shift in the *MKLN1* reading frame. *MKLN1* encodes the widely expressed intracellular protein muskelin 1, for which diverse functions in cell adhesion, morphology, spreading, and intracellular transport processes are discussed. While the pathogenesis of LAD remains unclear, our data facilitate genetic testing of Bull Terriers and Miniature Bull Terriers to prevent the unintentional production of LAD affected dogs. This study may provide a starting point to further clarify the elusive physiological role of muskelin 1 *in vivo*.

## Author summary

Lethal acrodermatitis (LAD) is an autosomal recessive hereditary disease in dogs. It is characterized by poor growth, immune deficiency and characteristic skin lesions of the paws and of the face. We mapped the LAD locus to a ~1.11 Mb segment on canine chromosome 14. Whole genome sequence data of an LAD affected dog and 191 controls revealed a candidate causative variant in the *MKLN1* gene, encoding muskelin 1. The identified variant, a single nucleotide substitution, *MKLN1*:c.400+3A>C, altered the 5’-splice site at the beginning of intron 4. We experimentally confirmed that this variant leads to complete skipping of exon 4 in the *MKLN1* mRNA in skin. Various cellular functions have been postulated for muskelin 1 including roles in intracellular transport processes, cell morphology, cell spreading, and cell adhesion. Our data from dogs reveal a novel *in vivo* role for muskelin 1 that is related to the immune system and skin. *MKLN1* thus represents a novel candidate gene for human patients with unsolved acrodermatitis and/or immune deficiency phenotypes. LAD affected dogs may serve as models to gain more insights into the function of muskelin 1.

## Introduction

Acrodermatitis enteropathica in humans (OMIM #201100) is an inherited disorder of zinc metabolism. Affected patients display an inflammatory rash, diarrhea and a general failure to thrive [1–3]. This disease is caused by variants in the *SLC39A4* gene encoding a zinc transporter that mediates the uptake of dietary zinc in the gut. Clinical signs in patients will ameliorate or even resolve upon oral supplementation with zinc [4]. A similar *SLC39A4* associated hereditary zinc deficiency exists in cattle [5].

**Fig 1:**
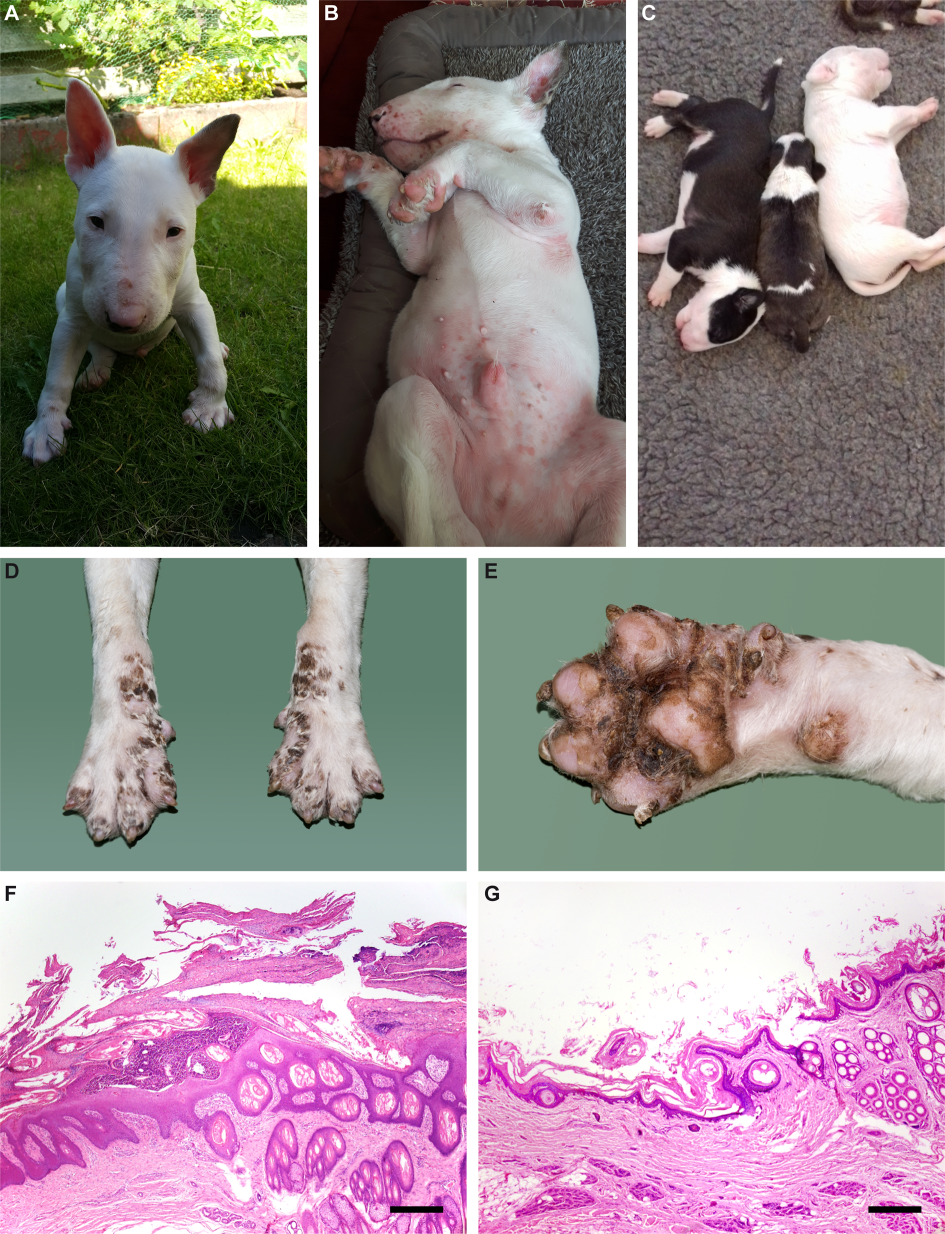
LAD phenotype. **(A)** Inflammatory skin lesions in the face of an affected Bull Terrier. **(B)** Similar lesions in the inguinal region. **(C)** LAD affected puppy in the middle of two non-affected littermates. A pronounced growth delay and a subtle coat color dilution are visible. **(D, E)** Fore paws of an LAD affected Bull Terrier puppy at necropsy. Symmetrical scaling and crusting of the skin including interdigital areas and foot pads is visible **(F, G)** Histopathological micrographs of the junction of interdigital haired skin and digital pad from an affected Bull Terrier puppy **(F)** and a control dog **(G)**. Marked thickening of the epidermis, excessive layers of non-cornifying epithelium and a large pustule are evident in the affected dog. Hematoxylin-eosin, bar = 400 µm.

In Bull Terriers, a related phenotype termed lethal acrodermatitis (LAD) has been reported in the scientific literature as early as 1986 [6]. LAD is inherited as a monogenic autosomal recessive trait. Affected puppies show characteristic skin lesions on the feet and on the face, diarrhea, bronchopneumonia, and a failure to thrive. The skin lesions consist of erythema and tightly adherent scales, erosions or ulcerations with crusts involving primarily the feet, distal limbs, elbows, hocks, and muzzle. Later on, hyperkeratosis of the footpads and deformation of the nails occur. LAD affected dogs also show a coat color dilution in pigmented skin areas. An abnormally arched hard palate impacted with decayed, malodorous food is a characteristic clinical marker for the disease (Fig 1) [6–8].

LAD dogs are immunodeficient with a reduction in serum IgA levels and frequently suffer from skin infections with *Malassezia* or *Candida* [9,10]. LAD manifests clinically in the first weeks of life. Affected puppies typically die before they reach an age of two years, either due to infections such as bronchopneumonia or because they are euthanized when their paw pad lesions become very severe and painful. They grow slower than their non-affected littermates and at the age of one year have about half the body weight and size of an unaffected dog [8]. Some, but not all studies found reduced levels of zinc in the serum of LAD affected dogs [6,8,11]. In contrast to acrodermatitis enteropathica in humans, oral or intravenous supplementation of zinc does not lead to an improvement of the clinical signs in LAD affected dogs [6]. A proteomic analysis reported changes related to inflammatory response in the liver of LAD affected puppies [12].

In the present study, we performed a genome-wide association study (GWAS) followed by a whole genome sequencing approach to unravel the causative genetic variant for LAD in Bull Terriers and Miniature Bull Terriers.

## Results

### Mapping of the LAD locus

**Fig 2:**
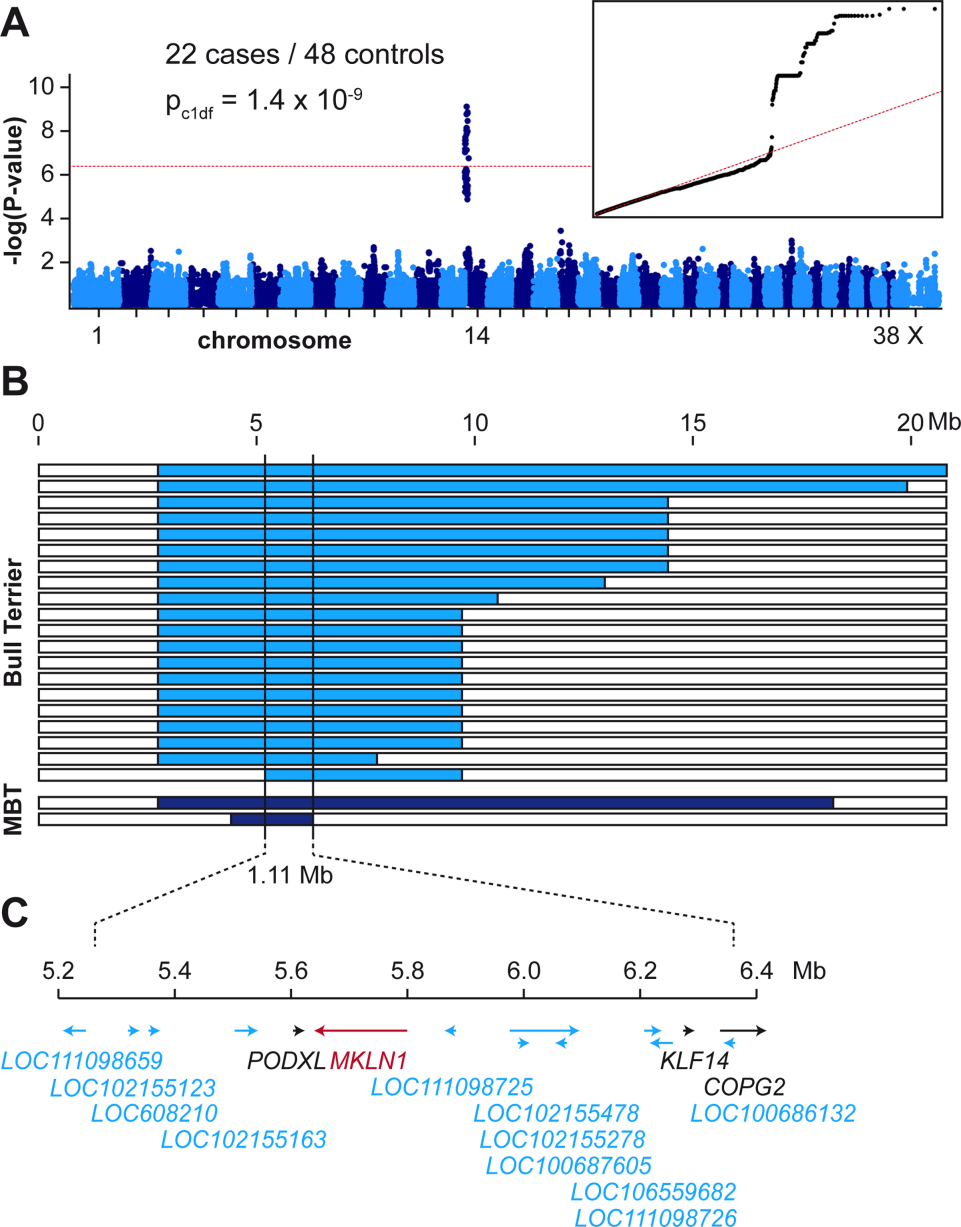
Mapping of the LAD locus. **(A)** A GWAS was performed in a cohort of 22 LAD cases and 48 controls. The Manhattan plot shows a single significant signal at the beginning of chromosome 14. The red line indicates the Bonferroni significance threshold (P_Bonf_ = 6.5 x 10^-7^). The quantile-quantile (QQ) plot in the inset shows the observed versus expected–log(p) values. The straight red line in the QQ plot indicates the distribution of p-values under the null hypothesis. The deviation of p-values at the right side indicates that these markers are stronger associated with the trait than it would be expected by chance. **(B)** Haplotype analysis in the 22 LAD cases. Each horizontal bar represents the chromosome 14 haplotypes of one dog. Twenty Bull Terriers and two Miniature Bull Terriers (MBT) had large homozygous intervals with allele sharing on chromosome 14 indicated in blue. The homozygous haplotype segment shared between all 22 dogs spanned ~1.11 Mb. The critical interval for the causative LAD variant corresponded to the interval between the first flanking heterozygous markers on either side or chr14:5,248,244–6,355,383 (CanFam 3.1 assembly). **(C)** Gene annotation for the critical interval. The NCBI annotation release 105 listed 4 protein coding genes (indicated in black or red) and 11 genes for non-coding RNAs (indicated in blue).

We performed a GWAS with genotypes from 78 Bull Terriers and Miniature Bull Terriers. After quality control, the pruned dataset consisted of 22 LAD cases, 48 controls and 76,419 markers. We obtained a single strong association signal with 57 markers exceeding the Bonferroni-corrected genome-wide significance threshold after adjustment for genomic inflation (P_Bonf._ = 6.5 x 10^−7^). All significantly associated markers were located on chromosome 14 within an interval spanning from 0.9 Mb– 10.6 Mb. The three top-associated markers all had a P-value of 1.4 x 10^−9^ and were located between 5.2 Mb– 5.9 Mb on chromosome 14 (Fig 2).

To narrow down the identified region, we visually inspected the genotypes of the cases to perform autozygosity mapping. We searched for homozygous regions with allele sharing and found one region of ~1.11 Mb, which was shared between all 22 cases. The critical interval for the causative LAD variant corresponded to the interval between the first flanking heterozygous markers on either side or chr14:5,248,244–6,355,383 (CanFam 3.1 assembly).

### Identification of a candidate causative variant

We sequenced the genome of an affected Bull Terrier at 24x coverage and called single nucleotide variants (SNVs) and small indel variants with respect to the reference genome (CanFam 3.1). We then compared these variants to whole genome sequence data of 3 wolves and 188 control dogs from genetically diverse breeds. This analysis identified five private homozygous variants in the critical interval in the affected dog (Table 1, S1 Table).

**Table 1:** Variants detected by whole genome re-sequencing of an LAD affected dog.

Filtering step^a^	Number of variants
Homozygous variants in the whole genome	3,061,192
Homozygous variants in the 1.11 Mb critical interval on chromosome 14	1,445
Private homozygous variants (absent from 191 control genomes) in critical interval	5

^a^ The sequences were compared to the reference genome (CanFam 3.1) from a Boxer.

Four of these five variants were intergenic and classified as “modifier” by the SNPeff software. The remaining fifth variant was located within the 5’-splice site of intron 4 of the *MKLN1* gene and its SNPeff impact prediction was “low”. The formal designation of this variant is chr14:5,731,405T>G or *MKLN1*:c.400+3A>C (S1 Fig).

**Fig 3:**
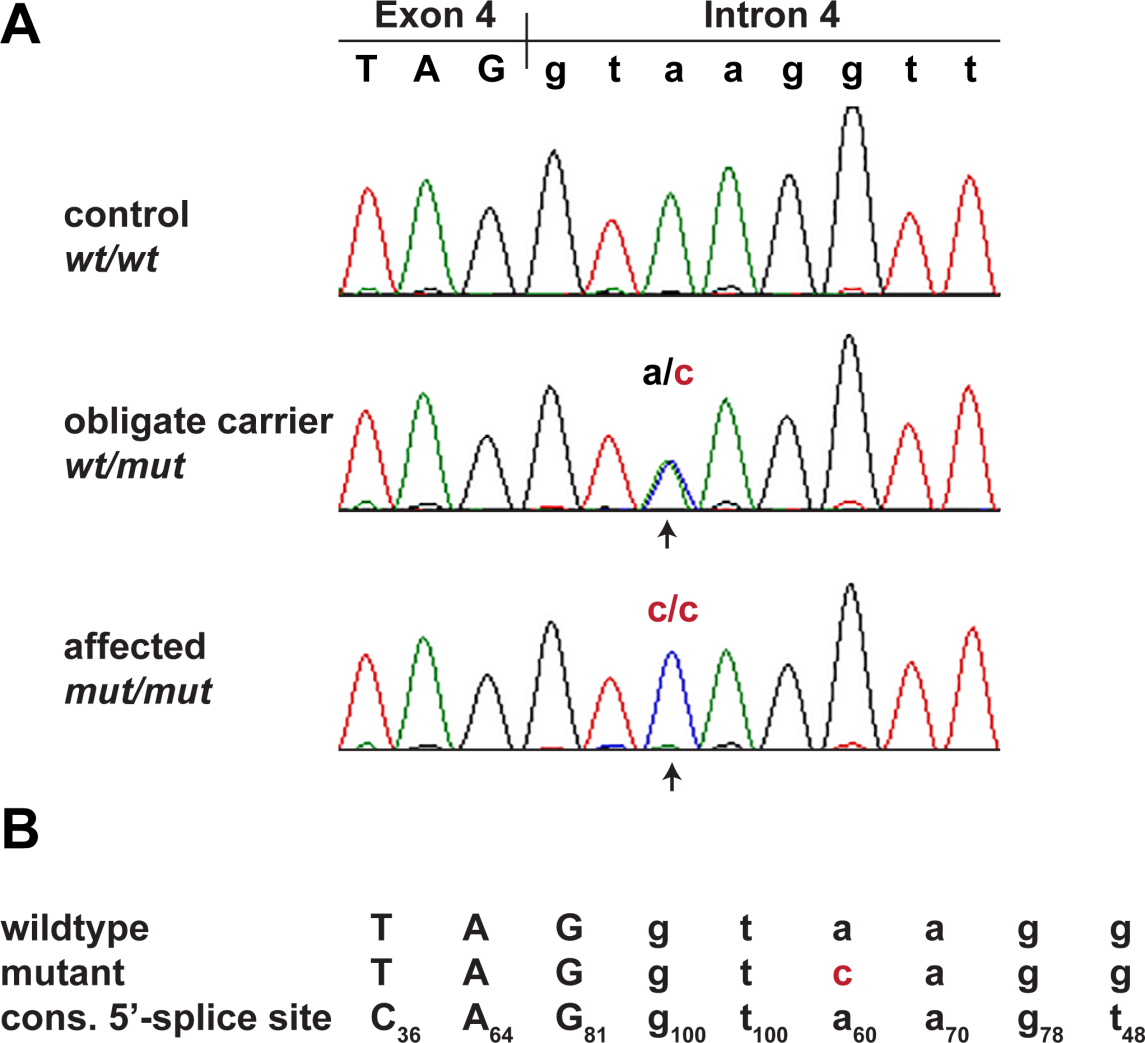
Sanger confirmation of the *MKLN1*:c.400+3A>C variant. **(A)** Electropherograms from dogs with the three different genotypes. **(B)** Wildtype and mutant allele compared to the consensus sequence for the human U2 GT-AG type 5’-splice sites [13]. Subscript numbers in the consensus sequence indicate the percentage of the respective conserved nucleotide in 183,682 investigated human 5’-splice site motifs of the U2 GT-AG type. The additional difference to the optimal consensus in the U1 spliceosomal RNA recognition site in the mutant allele is highlighted in red. In human 5’-splice sites the most frequent base at position 3 is an A (60%). G is also common at this position (35%), while C and T are both rare (<3%) [13].

We confirmed the presence of this variant by Sanger sequencing (Fig 3). As *MKLN1*:c.400+3A>C represented the only plausible candidate causative variant, we genotyped 251 Bull Terriers, 89 Miniature Bull Terriers, and 462 dogs from 62 other breeds for this variant (Table 2). The variant showed perfect association with the LAD phenotype in Bull Terriers and Miniature Bull Terriers (P_Fisher_ = 4.8 x 10^−58^). All 46 available cases were homozygous for the variant, whereas the unaffected dogs were either homozygous wildtype or heterozygous. The test dogs included a subset of unaffected Bull Terriers and Miniature Bull Terriers from Finland, which were not specifically collected for this study and therefore considered representative for the general population. The 166 Finnish dogs contained 37 heterozygous dogs (22%). The variant was not found in any of the tested dogs from other breeds.

**Table 2:** Association of the *MKLN1*:c.400+3A>C genotype with the LAD phenotype.

*MKLN1*:c.400+3A>C genotype	A/A	A/C	C/C
Cases, Bull Terrier (n = 41)	-	-	41
Cases, Miniature Bull Terrier (n = 5)	-	-	5
Controls, Bull Terrier (n = 210)	132	78	-
Controls, Miniature Bull Terrier (n = 84)	59	25	-
Controls, other breeds (n = 462)	462	-	-

### Functional confirmation

To assess the putative impact of the *MKLN1* variant on splicing, we analyzed the frequency of the wildtype and mutant sequence motifs in a compilation of 186,630 human 5’-splice sites [13,14]. The canine wildtype sequence TAGgtaagg was identical to the sequence of 276 human 5’-splice sites, while the mutant sequence motif TAGgtcagg occurred in only 3 human 5’-splice sites. The very low frequency of the mutant sequence motif suggested that *MKLN1*:c.400+3A>C might affect the efficacy of the splicing process. Several other pathogenic A>C transversions at 5’-splice sites’ position +3 with subsequent exon skipping have been described in the literature [15–17].

**Fig 4:**
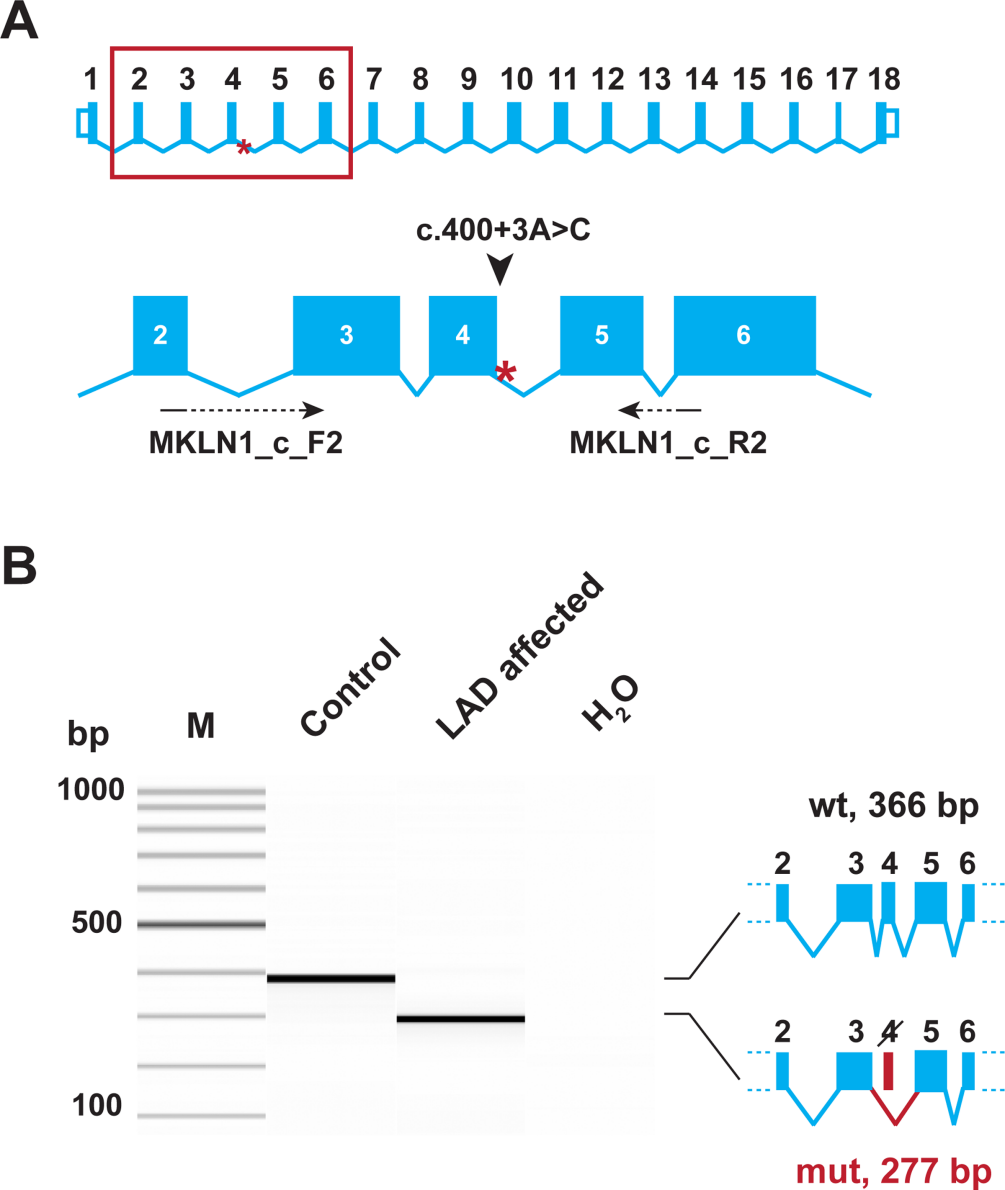
Experimental verification of the *MKLN1* splice defect. **(A)** The genomic organization of the *MKLN1* gene. Exons 2–6 are enlarged and the position of the primers used for RT-PCR is indicated. **(B)** RT-PCR was performed using skin cDNA from a control and an LAD affected Bull Terrier. The picture shows a Fragment Analyzer gel image of the experiment. In the control animal, only the expected 366 bp product is visible. In the LAD affected dog, a 277 bp product representing a transcript lacking exon 4 is visible. The identity of the bands was verified by Sanger sequencing. Thus, the *MKLN1*:c.400+3A>C variant leads to complete skipping of exon 4 (*MKLN1*:r.312_400del89).

We experimentally analyzed *MKLN1* transcripts in skin RNA from an LAD affected dog with the homozygous mutant C/C genotype in comparison to a healthy control dog (A/A genotype). RT-PCR with primers located at the exon 2/3 and exon 5/6 boundaries yielded a cDNA fragment of the expected size in the control dog, but not in the LAD affected dog. In the LAD affected dog, a very clean cDNA amplicon lacking exon 4 was obtained. This experiment demonstrated a complete skipping of exon 4 in *MKLN1* transcripts as consequence of the genomic *MKLN1*:c.400+3A>C variant (r.312_400del89; Fig 4). If translated, the mutant transcript was predicted to result in a severely truncated protein containing only the first 105 of a total of 735 amino acids of the wildtype protein (p.(Gly105SerfsTer10); S2 Fig).

## Discussion

In the present study we identified a splice defect in the canine *MKLN1* gene in Bull Terriers with LAD. The combination of GWAS and haplotype analysis localized the causative variant to a relatively small chromosomal region with only a few characterized genes including *MKLN1*. The splice region variant in *MKLN1* was the only plausible variant within this critical interval that showed the expected genotype concordance with the LAD phenotype in a large cohort of more than 300 Bull Terriers and ~500 dogs from other breeds.

The identified *MKLN1*:c.400+3A>C variant resulted in exon 4 skipping and a frameshift as 89 nucleotides were missing from the mutant transcripts. It therefore seems likely that mutant transcripts are degraded by nonsense-mediated mRNA decay. Considering the strong genetic association of the variant with the phenotype and the fact that we demonstrated a functional defect on the *MKLN1* transcript level, we think that our data strongly suggest the causality of the *MKLN1*:c.400+3A>C variant for LAD in Bull Terriers and Miniature Bull Terriers.

*MKLN1* encodes the widely expressed intracellular protein muskelin 1, also known as TWA2. The function of muskelin 1 is only partially understood. It was originally described as a protein that mediates adhesive and cell-spreading responses to thrombospondin 1, an extracellular matrix adhesion molecule [18]. However, different studies suggested that the function of muskelin 1 goes beyond this pathway and are also supported by the fact that muskelin 1, which has homologs in invertebrates and even fission yeast, evolved earlier than the vertebrate-specific thrombospondin 1 [19,20]. Muskelin 1 is a multidomain protein with an N-terminal discoidin domain, a LisH / CTLH tandem domain, and six C-terminal Kelch repeats, which forms homotetramers [21]. The LisH domain was shown to be crucial for muskelin 1 dimerization and cytoplasmic localization, and, together with the head-to-tail interaction via the discoidin domain, also for the tetramerization of muskelin 1 [20,21].

Consistent with its multidomain structure and ubiquitous expression, diverse binding partners have been reported for muskelin 1. It binds prostaglandin EP3 receptor isoform α [22] and heme-oxidase 1, which counteracts inflammatory and reactive oxygen species induced damage [23]. It is part of the CTLH complex, the homolog of yeast E3 ubiquitin ligase, where it binds to RanBPM and Twa I [24–26] and interacts with the cardiogenic transcription factor TBX-20 [27]. In the rat lens, muskelin 1 is a substrate of Cdk5 and interacts with the Cdk5 activator p39 [28]. Also in lens, it was shown that p39 links muskelin 1 to myosin II and stress fibers [29].

*Mkln1*^*-/-*^ knockout mice are viable and do not have skin lesions comparable to those in Bull Terriers with LAD. However, they exhibit a subtle coat color dilution phenotype similar to that seen in LAD affected dogs. In these *Mkln1*^*-/-*^ knockout mice, muskelin 1 was identified as a protein required for GABA_A_ receptor endocytosis and trafficking in neurons via direct interaction with the α1 subunit of GABA_A_ receptors and the motor proteins dynein and myosin VI. The dilute coat color of *Mkln1*^*-/-*^ knockout mice suggested that muskelin is a trafficking factor involved in several different intracellular transport processes, possibly including melanosome transport [30].

The lacking skin lesions in *Mkln1*^*-/-*^ knockout mice raise the questions whether muskelin 1 depletion does not result in disease in mice; whether their clinical signs would only manifest at a (much) older age; or whether the sterile environment of the laboratory animals prevented infections and thus the development of skin lesions. In the latter case, LAD would be a primary immunodeficiency disorder, in agreement with the observation of lower IgA levels and higher susceptibility to microbial infection in LAD affected dogs [8–10, 12]. Given the diverse known protein-protein interactions of muskelin 1, it is however likely that absence of muskelin 1 leads to dysfunctions beyond the immune system.

In humans, an intronic SNV in *MKLN1* was associated with urinary potassium excretion in Korean adults and another intronic *MKLN1* SNV with early bipolar disorder [31,32]. Furthermore, *MKLN1* has been associated with asthma in independent GWASs. A SNV in *MKLN1* ranked among the top 100 SNVs associated with childhood asthma in a study sample of 429 affected-offspring trios from a European American population [33]. A different SNV in the 5’-UTR of *MKLN1* was associated with asthma in a population including patients with severe or difficult-to-treat asthma [34].

In the ExAC database, only one *MKLN1* missense, but no nonsense, frameshift or splice site variants present in a homozygous state were found [35,36]. Furthermore, the probability of loss of function (LoF, specified as nonsense, splice acceptor, and splice donor variants) tolerance was estimated to be 1.00, indicating that the *MKLN1* gene is extremely LoF intolerant [37]. Therefore, it is conceivable that loss of function variants on both alleles might lead to severe phenotypes in humans.

To our knowledge, no link between muskelin 1 and zinc or copper metabolism has been reported to date. While acrodermatitis enteropatica in humans and acrodermatitis in cattle clinically resemble LAD in dogs, these diseases may be caused by completely different molecular mechanisms. The fact that findings on zinc levels in the few published studies on LAD affected dogs were contradictory and zinc supplementation did not lead to improvement of lesions [6] support this hypothesis.

In conclusion, we identified the *MKLN1*:c.400+3A>C variant leading to a splice defect in the *MKLN1* gene as candidate causative variant for LAD in Bull Terriers and Miniature Bull Terriers. The molecular pathogenesis of LAD remains unclear. Our data facilitate genetic testing of Bull Terriers and Miniature Bull Terriers to prevent the unintentional breeding of LAD affected dogs. LAD affected dogs may serve as models to further clarify the elusive physiological role of muskelin 1 *in vivo*.

## Materials and methods

### Ethics statement

All animal experiments were performed according to the local regulations. The dogs in this study were examined with the consent of their owners. The study was approved by the “Cantonal Committee For Animal Experiments” (Canton of Bern; permits 22/07, 23/10, and 75/16).

### Animals and samples

Bull Terriers with their characteristic egg-shaped head were founded as a dog breed in the 1850s in the United Kingdom. Originally, there were no size standards in this breed and smaller dogs were bred as a variety of the regular Bull Terrier. Eventually, two sub-populations formed and the Miniature Bull Terrier with a maximum height of 35.5 cm was recognized as an independent breed in 1991 by the American Kennel Club (AKC) and in 2011 by the European Fédération Cynologique Internationale (FCI). Therefore, Bull Terriers and Miniature Bull Terriers share a common ancestral gene pool, but represent independent closed populations today.

This study included samples from 251 Bull Terriers (41 LAD cases / 210 controls) and 89 Miniature Bull Terriers (5 LAD cases / 84 controls). Case/controls status was based on owners’ reports. We additionally used 462 dogs from 62 breeds, which were assumed to be free of the disease allele (S3 Table). Skin biopsies were taken from two LAD affected Bull Terriers from toe, nose, lip, and forearm and fixed in 10% buffered formalin for 24 hours. Biopsies were processed, embedded in paraffin and sectioned at 4 µm. Skin sections were stained with hematoxylin and eosin. The histopathology was performed by veterinary pathologists (BR, Dipl.-ECVP, and SH). Two further biopsies from comparable sites of the same dogs were submerged in RNAlater solution for subsequent RNA isolation.

### DNA isolation and SNV genotyping

We isolated genomic DNA from EDTA blood samples. Seventy-eight dogs were genotyped for either 173,662 or 218,256 SNVs on the illumina canine_HD chip. The raw SNV genotypes are available at https://www.animalgenome.org/repository/pub/BERN2017.1208/.

### GWAS

The initial dataset consisted of 78 dogs and 220,853 markers. Using Plink version 1.9 [38] we excluded markers that were not located on autosomes or the X chromosome (n = 2,327) and markers with a genotyping rate lower than 90% (n = 49,814). Using the R package GenABEL [39] and the command “check.markers”, dogs with a call rate < 90% (n = 3), ibs > 95% (n = 0), high individual heterozygosity (FDR = 0.01) (n = 1, included in dogs with low call rate) as well as markers with a maf < 1% (n = 55,931) and a genotyping rate < 90% (n = 10,951) were excluded. Five outliers in the multidimensional scaling plot based on a genomic distance matrix were also removed. In a second quality control step, markers deviating from Hardy-Weinberg equilibrium (FDR = 0.2) in controls (n = 1,239), markers with a genotyping rate < 90% (n = 0) and maf < 1% (n = 26,215) were excluded, resulting in a final dataset of 70 dogs (22 cases, 48 controls) and 76,419 markers. A polygenic model of the hglm package [40], with a kinship matrix based on autosomal markers in the cleaned dataset as random effect, was estimated and a score test for association using the function “mmscore” was performed. The genomic inflation factor was 1.16. We corrected for multiple testing using Bonferroni correction with a significance level of 0.05. QQ plots were created using qqman version 0.1.4 [41].

### Haplotype analysis

We visually inspected plink tped files for the region of interest on chromosome 14 using Excel and searched for homozygous regions with haplotype sharing in cases with a call rate >90%. The first flanking heterozygous markers on either side of the homozygous region in 22 cases defined the borders of the critical interval.

### Whole genome sequencing of an affected Bull Terrier

An Illumina PCR-free TruSeq fragment library with 350 bp insert size of an LAD affected Bull Terrier was prepared. We collected 219 million 2 x 150 bp paired-end reads or 24x coverage on a HiSeq3000 instrument. The reads were mapped to the dog reference genome assembly CanFam3.1 and aligned using Burrows-Wheeler Aligner (BWA) version 0.7.5a [42] with default settings. The generated SAM file was converted to a BAM file and the reads were sorted by coordinate using samtools [43]. Picard tools (http://sourceforge.net/projects/picard/) was used to mark PCR duplicates. To perform local realignments and to produce a cleaned BAM file, we used the Genome Analysis Tool Kit (GATK version 2.4.9, 50) [44]. GATK was also used for base quality recalibration with canine dbsnp version 139 data as training set. The sequence data were deposited under the study accession PRJEB16012 and sample accession SAMEA4504844 at the European Nucleotide Archive.

### Variant calling

Putative SNVs were identified in each of 192 samples (S2 Table) individually using GATK HaplotypeCaller in gVCF mode [45]. Subsequently all sample gVCF files were joined using Broad GenotypeGVCFs walker (-stand_emit_conf 20.0; -stand_call_conf 30.0). Filtering was performed using the variant filtration module of GATK using the following standard filters: SNVs: Quality by Depth: QD < 2.0; Mapping quality: MQ < 40.0; Strand filter: FS > 60.0; MappingQualityRankSum: MQRankSum < -12.5; ReadPosRankSum < -8.0. INDELs: Quality by Depth: QD < 2.0; Strand filter: FS > 200.0. The functional effects of the called variants were predicted using SnpEFF software [46] together with the NCBI annotation release 104 on CanFam 3.1. For the filtering of candidate causative variants in the case, we used 191 control genomes, which were either publicly available [47] or produced during other projects of our group or contributed by members of the Dog Biomedical Variant Database Consortium. A detailed list of these control genomes is given in S2 Table.

### Gene analysis

We used the dog CanFam 3.1 reference genome assembly for all analyses. Numbering within the canine *MKLN1* gene corresponds to the accessions XM_005628367.3 (mRNA) and XP_005628424.1 (protein). Numbering within the human *MKLN1* gene corresponds to the accessions NM_013255.4 (mRNA) and NP_037387.2 (protein).

### Sanger sequencing

We used Sanger sequencing to confirm the candidate variant *MKLN1*:c.400+3A>C and to genotype the dogs in this study. A 797 bp fragment containing the variant was PCR amplified from genomic DNA using AmpliTaq Gold 360 Master Mix (Life Technologies) and the primers CCATGCACTGTAGCCACATC and TGGAAAAGGTTCCACTTGAAAT. After treatment with shrimp alkaline phosphatase and endonuclease I, PCR products were directly sequenced on an ABI 3730 capillary sequencer (Life Technologies). We analyzed the Sanger sequence data using the software Sequencher 5.1 (GeneCodes).

### RNA isolation and RT-PCR

RNA was extracted from skin samples using the RNeasy Fibrous Tissue Mini Kit (Qiagen). The tissue was first finely crushed by mechanical means using TissueLyser (Quiagen), and RNA was extracted by centrifugation following the instructions by the manufacturer. Total mRNA was reverse transcribed into cDNA using the SuperScript IV Reverse Transcriptase kit (Thermo Fisher) with oligo d(T) primers. A PCR on the synthesized cDNA was carried out using primer MKLN1_c_F2, CCTCCCCAGTACTTGATTCTG, located at the boundary of exons 2 and 3, and primer MKLN1_c_R2, TTCCTGTTCACGGTACTTGC, located at the boundary of exons 5 and 6 of the *MKLN1* gene. The products were analyzed on a Fragment Analyzer capillary gel electrophoresis instrument (Advanced Analytical). The sequence of the obtained RT-PCR products was confirmed by Sanger sequencing as described above.

## Supporting information

S1 Fig Sequence context of the *MKLN1* variant. (PDF)

S2 Fig Predicted amino acid sequences of the wildtype and mutant MKLN1 proteins. (PDF)

S1 Table Private variants in the critical interval. (XLSX)

S2 Table Control dogs used for whole genome sequencing. (XLSX)

S3 Table Control dogs genotyped for the *MKLN1* variant. (XLSX)
