# Supplementary material for: MKLN1 splicing defect in dogs with lethal acrodermatitis
Source: PLoS Genet. 2018 Mar 22;14(3):e1007264. doi: 10.1371/journal.pgen.1007264 (PMC5863938; doi:10.1371/journal.pgen.1007264)
Supplement: S1 Fig — (PDF) [file pgen.1007264.s001.pdf]

S1 Figure: Sequence context of the *MKLN1* variant

Genetic variant:	Chr14:5,731,405T>G	(CanFam 3.1 assembly)
	MKLN1:c.400+3A>C	(XM_005628367.3)
	MKLN1:r.312_400del	(XM_005628367.3)

The sequence below represents the reverse complement of positions 5,730,921 – 5,732,000, chromosome 14 genomic sequence, derived from Genbank accession NC_006596.3 (CanFam3.1 assembly). Intronic sequences are given in lowercase letters. Exonic sequences are given in capital letters. The c.400+3A>C variant indicated in red leads to skipping of the preceding exon 4. Primers used for PCR amplification of a 797 bp product and genotyping are indicated beneath the sequence.

```
5,732,000     tgtttatgta gagaagtgac atgggtttat ttttatttag gatgaaatgg gtgctattaa     5,731,941
5,731,940     accaatggag tgtttattga accgtaaaag gaacattatt gacatcataa tcagaatttt     5,731,881
5,731,880     gaaaaactta attgtttcac agactaatat ttggctctga gtcgttagat aggaaagtaa     5,731,821
5,731,820     attattttaa tttttttctt actgcttaca tattttatgt ttatatcctg ttatacacta     5,731,761
5,731,760     aggacaccag gtactatcac acatttccat gcactgtagc cacatccttt aattaacatt     5,731,701
                                          >>>>>>>>>>>>>>>>>>>>>>>>

5,731,700     gaacattttt cattaagcct tattatataa ttaggagagt acagttataa tgcagttgat     5,731,641
5,731,640     atgaatgtag aacttttgac ctatgttctc atgtttgact acttctttac attcttttgg     5,731,581
5,731,580     tcttgtagtt tgtctaacct aaagatctga gcatgattta tgtttactaa attataaaat     5,731,521
5,731,520     cagataacct atttttttc acagTGGCTT AAAGAATGAT TATAACAAAG AAACATTCAC     5,731,461

                                                               c.400+3A>C ↓
5,731,460     CTTGAAGCAT AAAATTGATG AACAGATGTT CCCTTGTCGA TTCATTAAAA TAGgtaaggt     5,731,401
5,731,400     tttagcattc tgaaatagaa ataaattctt tataccacca ctcagaaaac taaagcctgt     5,731,341
5,731,340     gggctgaatc cttcagaact tctgtttttt aaaataagat tttatgggaa cactaccaca     5,731,281
5,731,280     ctgattatta actggttata atgtctatgt cttgcatttg ctctacaaag tagttgcaag     5,731,221
5,731,220     agacaaaatg tttactgttt ggccctctgc atcaaaagtt tactgttccc tgggcagccc     5,731,161
5,731,160     cggtggctca gtggtttagc gccgccctca gcccagggcg tgatcctgga gacccaggac     5,731,101
5,731,100     ggagtcccat gtcaggctcc ctgcatggag cctgtttctc cctccgcctg tctctctctg     5,731,041
5,731,040     actctaataa ataaataaaa tcttaaaaaa aaaaaaaaaa ggtttctgtt ccctgcttta     5,730,981
5,730,980     caagtttata agtgatattt atttcaagtg gaacctttc caaatatttt ttcatttgaa     5,730,921
                                    <<<<<<<<<<<<<<<<<<<<<<<
```
