# Supplementary material for: MKLN1 splicing defect in dogs with lethal acrodermatitis
Source: PLoS Genet. 2018 Mar 22;14(3):e1007264. doi: 10.1371/journal.pgen.1007264 (PMC5863938; doi:10.1371/journal.pgen.1007264)
Supplement: S2 Fig — (PDF) [file pgen.1007264.s002.pdf]

S2 Figure: Predicted amino acid sequences of the wildtype and mutant *MKLN1* proteins

Genetic variant:	Chr14:5,731,405T>G	(CanFam 3.1 assembly)
	MKLN1:c.400+3A>C	(XM_005628367.3)
	MKLN1:r.312_400del	(XM_005628367.3)
	MKLN1:p.(Gly105SerfsTer10)	(XP_005628424.1)

```
wildtype    maaggavaaa pecrllpyal hkwssfssty lpenilvdkp ndqssrwsse snyppqylil    60       1..160 discoidin
            |||||||||| |||||||||| |||||||||| |||||||||| |||||||||| ||||||||||                    domain
mutant      maaggavaaa pecrllpyal hkwssfssty lpenilvdkp ndqssrwsse snyppqylil    60

wildtype    klerpaivqn itfgkyekth vcnlkkfkvf ggmneenmte llssglkndy nketftlkhk   120
            |||||||||| |||||||||| |||||||||| |||||||||| ||||
mutant      klerpaivqn itfgkyekth vcnlkkfkvf ggmneenmte llsssalivg tql          113

wildtype    ideqmfpcrf ikivpllswg psfnfsiwyv elsgiddpdv vqpclnwysk yreqeairlc   180     172..204 LisH domain
wildtype    lkhfrqhnyt eafeslqkkt kialehpmlt dlhdklvlkg dfdaceelxe kavndglfnq   240     206..258 CTLH domain
wildtype    yisqqeykpr wsqiipkstk gdgednrpgm rgghqmvidv qtetvylfgg wdgtqdladf   300     284..330 Kelch 1
wildtype    waysvkenqw tcisrdteke ngpsarschk mcidiqrrqi ytlgryldss vrnskslksd   360     339..391 Kelch 2
wildtype    fyrydidtnt wmllsedtaa dggpklvfdh qmcmdsekhm iytfggrilt cngsvddsra   420     408..458 Kelch 3
wildtype    sepqfsglfa fncqcqtwkl lredscnagp ediqsrighc mlfhsknrcl yvfggxrskt   480     469..515 Kelch 4
wildtype    ylndffsydv dsdhvdiisd gtkkdsgmvp mtgftqrati dpelneihvl sglskdkekr   540     526..578 Kelch 5
wildtype    eenvrnsfwi ydivrnswsc vykndqaakd npskslqeee pcprfahqlv ydelhkvhyl   600     597..651 Kelch 6
wildtype    fggnpgkscs pkmrlddfws lklcrpskdy llrhckylir khrfeekaqm dplsalkylq   660
wildtype    ndlyitvdhs dpeetkefql lasalfksgs dftalgfsdv dhtyaqrtql fdtlvnffpd   720
wildtype    smtppkgnlv dlitl                                                    735
```

An alignment of the canine wildtype MKLN1 protein with the predicted translation product of the mutant transcript is shown. Skipping of exon 4 leads to a frameshift and an early premature stop codon. The predicted mutant protein lacks 630 amino acids (86%) of the wildtype protein. The wildtype canine MKLN1 protein has the same length of 735 amino acids as the human MKLN1 protein (NP_037387.2) and differs in only 6 amino acids from its human homolog, which corresponds to 99.2% sequence identity. Important functional domains of MKLN1 are highlighted and annotated.
